# A multimodal dataset of human gait at different walking speeds established on injury-free adult participants

**DOI:** 10.1038/s41597-019-0124-4

**Published:** 2019-07-03

**Authors:** Céline Schreiber, Florent Moissenet

**Affiliations:** Centre National de Rééducation Fonctionnelle et de Réadaptation – Rehazenter, Laboratoire d’Analyse du Mouvement et de la Posture, Luxembourg, Luxembourg

**Keywords:** Biomedical engineering, Motor cortex

## Abstract

Human motion capture is used in various fields to analyse, understand and reproduce the diversity of movements that are required during daily-life activities. The proposed dataset of human gait has been established on 50 adults healthy and injury-free for lower and upper extremities in the most recent six months, with no lower and upper extremity surgery in the last two years. Participants were asked to walk on a straight level walkway at 5 speeds during one unique session: 0–0.4 m.s^−1^, 0.4–0.8 m.s^−1^, 0.8–1.2 m.s^−1^, self-selected spontaneous and fast speeds. Three dimensional trajectories of 52 reflective markers spread over the whole body, 3D ground reaction forces and moment, and electromyographic signals were simultaneously recorded. For each participants, a minimum of 3 trials per condition have been made available in the dataset for a total of 1143 trials. This dataset could increase the sample size of similar datasets, lead to analyse the effect of walking speed on gait or conduct unusual analysis of gait thanks to the full body markerset used.

## Abstract (summary)

**Table:** 

Design Type(s)	modeling and simulation objective • data integration objective
Measurement Type(s)	gait • force • muscle electrophysiology trait
Technology Type(s)	digital camera • force transducer • electromyography
Factor Type(s)	speed • sex • age • height • weight • body mass index (BMI) • limb length
Sample Characteristic(s)	Homo sapiens • whole body

Machine-accessible metadata file describing the reported data (ISA-Tab format)

## Background & Summary

Human motion capture is nowadays commonly used in various fields to analyse, understand and reproduce the diversity of movements that can be produced during daily-life activities. In clinical practice, the emergence of evidence-based medicine promoted the development of quantitative assessment tools for the diagnosis and treatment of pathology-related movement disorders. In particular, the process of gait disorders analysis currently often consists of the measurement of joint kinematics and kinetics in three dimensions^1^. This assessment is called clinical gait analysis (CGA) and attempts to provide an objective record that quantifies the magnitude of deviations from normal gait^2^. On this basis, a set of pathology-related impairments having the most impact on gait is identified and can be used to target the treatment^3^.

However, the identification of deviations is highly dependent with the characteristics of the normative database used^4^. Special attention is then required to discriminate the differences between pathological and asymptomatic populations that could confound deviations. In particular, the gait of pathological populations is often observed at their own self-selected walking speed and compared to normative data established at the spontaneous walking speed of an asymptomatic population^5^. Since the spontaneous walking speed of pathological populations (*e*.*g*. ranged between 0.18 and 1.03 m.s^−1^ for stroke^6^) is often slower than for an asymptomatic population (ranged between 1.04 and 1.60 m.s^−1^ ^7^), a walking speed mismatch appears. Because walking speed is known to affect kinematics, kinetics, spatiotemporal parameters and muscular activity^8^, the identification of gait deviations can then become challenging since both pathology and walking speed difference may contribute to them^9^. But walking speed is not the only variable that could be source of a mismatch in comparison of a patient and an asymptomatic population. Demographic and anthropometric parameters may also affect CGA interpretation. Recently, Chehab *et al*.^10^ demonstrated the impact of walking speed, but also age, sex and body mass index (BMI) on 3D kinematics and kinetics of the lower limb during gait. While walking speed was the most influential variable, the authors highlighted the influence of demographic and anthropometric parameters on very common parameters (*e*.*g*. pelvis tilt, peak of hip extension) used in the identification of gait deviations.

Several datasets have been made available in the literature and can be used to ease the establishment of a broad normative database allowing to match patient characteristics^11–14^. However, few datasets include all the common parameters on a large number of subjects (*i*.*e*. spatio-temporal, kinematics, kinetics, electromyography signals). The proposed dataset has been established on 50 healthy participants aged between 19 and 67 years. They were asked to walk on a straight level walkway at five different walking speeds: between 0 and 0.4 m.s^−1^, between 0.4 and 0.8 m.s^−1^, between 0.8 and 1.2 m.s^−1^, self-selected spontaneous speed and self-selected fast speed. Three dimensional trajectories of 52 cutaneous reflective markers spread over the whole body, 3D ground reaction forces and moment, and electromyographic signals were simultaneously recorded. For each participant, 3 trials for each walking speed condition plus one static were recorded and pre-processed, for a total of 1143 trials. This dataset could increase population sample size of similar datasets, lead to analyse the effect of walking speed on gait or conduct unusual analysis of gait characteristics thanks to the full body markerset used.

## Methods

### Participants

Fifty participants (24 women and 26 men, 37.0 ± 13.6 years, 1.74 ± 0.09 m, 71.0 ± 12.3 kg) were recruited on a voluntary basis. The study was approved by the institutional medical ethic committee of the Rehazenter and follows the recommendations of the declaration of Helsinki. The participants gave their informed consent to participate in the study. All participants were asymptomatic, *i*.*e*. healthy and injury free for both lower and upper extremities in the most recent six months, and no lower or upper extremity surgery in the last two years. Furthermore, only participants having a leg length difference lower than 1.5% of the height (corresponding to a maximum of 0.03 m) were included in this study to avoid an effect of a leg length discrepancy in the dataset.

### Procedure

For each participant, the entire data collection was acquired in a single session which lasted approximately 2 hours. All the sessions were managed by the same experienced operator. The following procedure was adopted:

1. *Calibration of the systems*: This calibration was performed following the instructions available in the manufacturer’s documentation, including the definition of the inertial coordinate system, the dynamic calibration of the cameras, and the zeroing of forceplates.
2. *Introduction to the participant*: The operator introduced the laboratory, outlined the need to establish the database, and briefly explained the conduct of the session, including the material used. The participant could ask questions at any time.
3. *Interview*: An interview allowed collecting information at this stage about participant’s health condition and sports habits (Supplementary Table 1).
4. *Preparation of the participant*: The participant was asked to change clothes to tight-fitting clothes or underwear, including removing shoes and socks as the acquisition was barefoot, and tied up their hair if necessary. The operator also collected participants’ anthropometric and demographic information (Online-only Table 1). The participant was then equipped with EMG electrodes and cutaneous reflective markers (see section *Records*).
5. *Static record*: The participant was standing upright with lower and upper limbs outstretched, palms facing forward, right head with straight eyes. Five seconds without any movement were recorded. The record was verified by the operator. A new standing trial was performed if any marker was missing or movements perturbed the record.
6. *Walking trials*: The participant was asked to walk back and forth on a 10-m straight level walkway. The instruction given was “to walk as naturally as possible, looking forward”. No directive was given about the forceplates to avoid a conscious adaptation of the walk. A minimum of 3 trials were recorded for each condition. All trials were rapidly verified by the operator. Five conditions of walking speed were recorded: between 0 and 0.4 m.s^−1^ (C1), between 0.4 and 0.8 m.s^−1^ (C2), between 0.8 and 1.2 m.s^−1^ (C3), self-selected spontaneous speed (C4) and self-selected fast speed (C5). Conditions C1, C2 and C3 were induced by a metronome^15^ and correspond to the three groups described by Perry^16^ (*i*.*e*. household ambulators, limited community ambulators and community ambulators). An adaptation time to the imposed cadence was foreseen for these 3 conditions and the velocity of the first trial was checked to be in the expected range of speed. C4 and C5 were self-selected conditions in response to the instructions to walk respectively “as usual” and “fast but not running”.
7. *Session ending*: All markers and electrodes were removed. Additional explanations about the records were given to the participants while showing some videos and 3D animations.

### Records

**Fig. 1:**
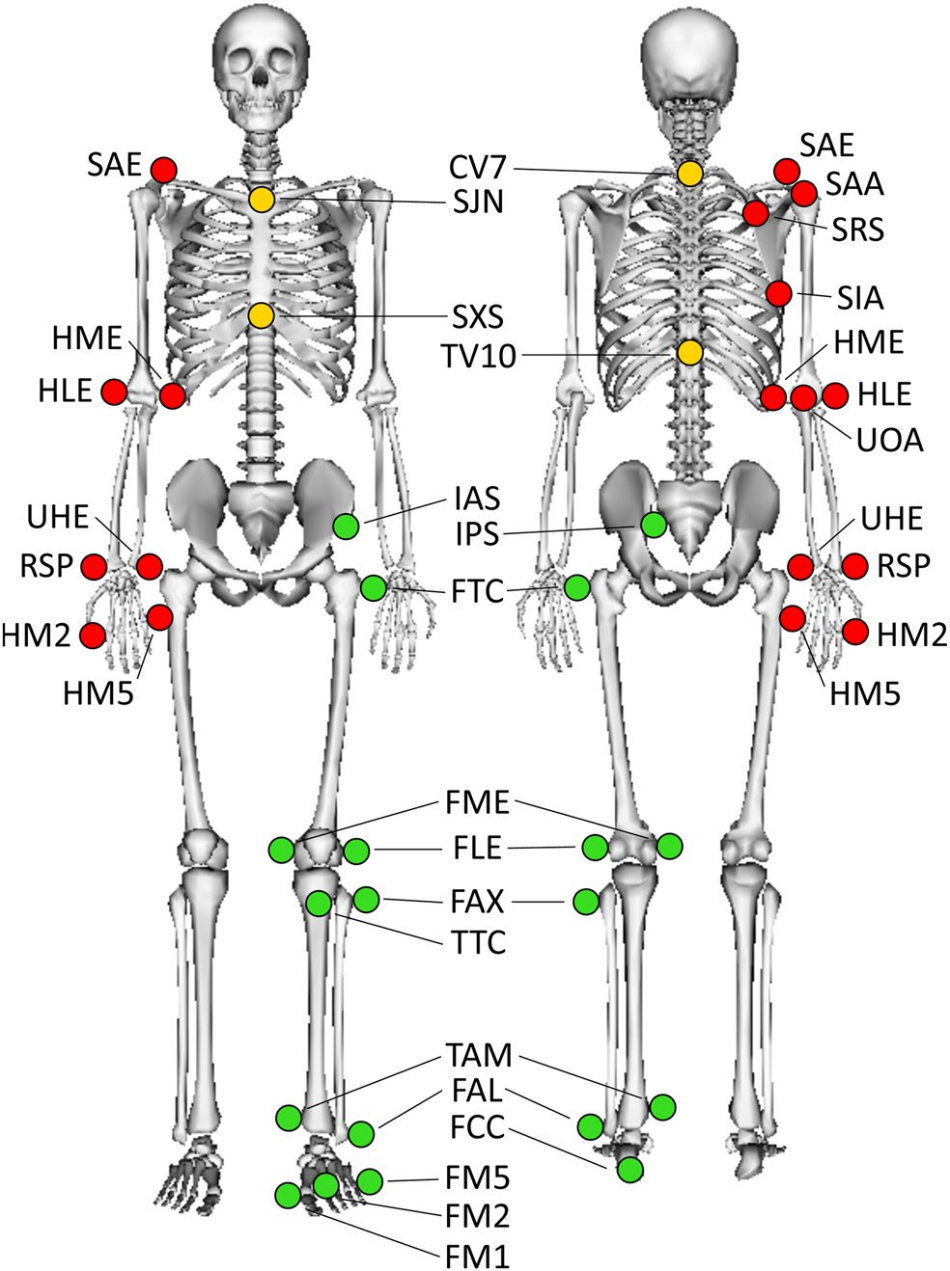
Reflective cutaneous markers placed by anatomical palpation on the participants. Only left side markers have been illustrated for the lower limbs (green markers) and right side markers for the upper limbs (red markers). The anatomical description and full name of each marker are given in Table 1.

A 10-camera optoelectronic system sampled at 100 Hz (OQUS4, Qualisys, Sweden) was used to track the three-dimensional (3D) trajectories of a set of 52 cutaneous reflective markers. The markerset (Fig. 1, Table 1) was defined to allow the use of the biomechanical model proposed by Dumas and Wojtusch^17^. This model follows the recommendations of the International Society of Biomechanics (ISB)^18,19^ for the definitions of joint coordinate systems and joint centres. Marker placement was achieved by anatomical palpation (anatomical landmarks reported in Table 1) following the guideline provided by Van Sint Jan^20^ and remained unchanged during all trials. The same experienced physiotherapist performed both anatomical palpation and marker placement on all included participants. Two forceplates sampled at 1500 Hz (OR6-5, AMTI, USA) were used to record 3D ground reaction force and moment. These forceplates were embedded in the middle of the walkway travelled during the overground walking trials. A wireless electromyographic (EMG) system sampled at 1500 Hz (Desktop DTS, Noraxon, USA) was used to record the EMG signals collected by 8 probes connected to pairs of surface electrodes with a diameter of 10 mm (Ambu Neuroline 720, Ambu, Denmark). Skin preparation, inter-electrode distance, and electrode locations followed the recommendations of the Surface Electromyography for the Non-Invasive Assessment of Muscles (SENIAM) project^21^. Skin preparation consisted in cleaning with alcohol, preceded by shaving, when necessary. An inter-electrode distance of 20 mm was applied for each muscle. EMG signals were recorded on 8 muscles of the right leg: gluteus maximus, gluteus medius, rectus femoris, vastus medialis, semitendinosus, gastrocnemius medialis, soleus, and tibialis anterior. In order to reduce the baseline noise contamination due to movement artefacts, each probe with related cables and electrodes were maintained using a self-adherent wrap (Coban, 3 M, USA). All these systems were synchronised using the Qualisys Track Manager software (QTM 2.8.1065, Qualisys, Sweden).

**Table 1:** Marker trajectories stored in c3d files.

Labels	Format	Dim.	Unit	Description
L_IAS	Real	n* × 3	mm	Left anterior-superior iliac spine coordinates
L_IPS	Real	n × 3	mm	Left posterior-superior iliac spine coordinates
R_IPS	Real	n × 3	mm	Right posterior-superior iliac spine coordinates
R_IAS	Real	n × 3	mm	Right anterior-superior iliac spine coordinates
L_FTC	Real	n × 3	mm	Left greater trochanter coordinates
L_FLE	Real	n × 3	mm	Left lateral femoral epicondyle coordinates
L_FME	Real	n × 3	mm	Left medial femoral epicondyle coordinates
L_FAX	Real	n × 3	mm	Left fibula head coordinates
L_TTC	Real	n × 3	mm	Left tibial tuberosity coordinates
L_FAL	Real	n × 3	mm	Left lateral tibial malleolus coordinates
L_TAM	Real	n × 3	mm	Left medial tibial malleolus coordinates
L_FCC	Real	n × 3	mm	Left posterior calcaneus coordinates
L_FM1	Real	n × 3	mm	Left 1^st^ metatarsal head coordinates
L_FM2	Real	n × 3	mm	Left 2^nd^ metatarsal head coordinates
L_FM5	Real	n × 3	mm	Left 5^th^ metatarsal head coordinates
R_FTC	Real	n × 3	mm	Right greater trochanter coordinates
R_FLE	Real	n × 3	mm	Right lateral femoral epicondyle coordinates
R_FME	Real	n × 3	mm	Right medial femoral epicondyle coordinates
R_FAX	Real	n × 3	mm	Right fibula head coordinates
R_TTC	Real	n × 3	mm	Right tibial tuberosity coordinates
R_FAL	Real	n × 3	mm	Right lateral tibial malleolus coordinates
R_TAM	Real	n × 3	mm	Right medial tibial malleolus coordinates
R_FCC	Real	n × 3	mm	Right posterior calcaneus coordinates
R_FM1	Real	n × 3	mm	Right 1^st^ metatarsal head coordinates
R_FM2	Real	n × 3	mm	Right 2^nd^ metatarsal head coordinates
R_FM5	Real	n × 3	mm	Right 5^th^ metatarsal head coordinates
CV7	Real	n × 3	mm	7^th^ cervical vertebra coordinates
TV10	Real	n × 3	mm	Spinous process of the 10^th^ thoracic vertebrae coord.
SXS	Real	n × 3	mm	Suprasternal notch coordinates
SJN	Real	n × 3	mm	Xiphoid process coordinates
L_SIA	Real	n × 3	mm	Left acromial tip coordinates
L_SRS	Real	n × 3	mm	Left spine root coordinates
L_SAA	Real	n × 3	mm	Left acromial angle coordinates
L_SAE	Real	n × 3	mm	Left acromial edge coordinates
L_HLE	Real	n × 3	mm	Left lateral humerus epicondyle coordinates
L_HME	Real	n × 3	mm	Left medial humerus epicondyle coordinates
L_UOA	Real	n × 3	mm	Apex of the left olecranon coordinates
L_RSP	Real	n × 3	mm	Left radius styloid process coordinates
L_UHE	Real	n × 3	mm	Left ulnar styloid process coordinates
L_HM2	Real	n × 3	mm	Left head of the 2^nd^ metacarpus coordinates
L_HM5	Real	n × 3	mm	Left head of the 5^th^ metacarpus coordinates
R_SIA	Real	n × 3	mm	Right acromial tip coordinates
R_SRS	Real	n × 3	mm	Right spine root coordinates
R_SAA	Real	n × 3	mm	Right acromial angle coordinates
R_SAE	Real	n × 3	mm	Right acromial edge coordinates
R_HLE	Real	n × 3	mm	Right lateral humerus epicondyle coordinates
R_HME	Real	n × 3	mm	Right medial humerus epicondyle coordinates
R_UOA	Real	n × 3	mm	Apex of the right olecranon coordinates
R_RSP	Real	n × 3	mm	Right radius styloid process coordinates
R_UHE	Real	n × 3	mm	Right ulnar styloid process coordinates
R_HM2	Real	n × 3	mm	Right head of the 2^nd^ metacarpus coordinates
R_HM5	Real	n × 3	mm	Right head of the 5^th^ metacarpus coordinates

*Number of frames recorded at 100 Hz.

### Data processing

Labelling of the marker trajectories was performed in the Qualisys Tracking Manager software (QTM 2.8.1065, Qualisys, Sweden) and all foot strike and foot off events were manually detected by the same experienced operator. Events were defined based on the threshold of 5 N applied on the vertical ground reaction force, or based on markers trajectories when ground reaction forces were not available. Raw marker trajectories, ground reaction forces and moments and EMG signals, as well as time events, were then exported in the standard c3d file format (https://www.c3d.org) and then imported and processed under Matlab (R2018a, The MathWorks, USA) using the Biomechanics ToolKit (BTK)^22^. Markers trajectories (expressed in mm) were interpolated when necessary using a reconstruction based on marker inter-correlations obtained from a principal component analysis^23^. Then, trajectories were smoothed using a 4^th^ order Butterworth low pass filter with a 6 Hz cut-off frequency. Ground reaction forces and moments (expressed in N and N.mm, respectively) were smoothed using a 2^th^ order Butterworth low pass filter with a 15 Hz cut-off frequency. Below the threshold of 5 N defined on the vertical ground reaction force, all of these forces and moments were set to zero. EMG signals (expressed in V) were band pass filtered between 30 and 300 Hz (4^th^ order Butterworth filter) to reduce artefacts due to motion and electromagnetic fields. All processed data were cropped few frames before the first event and few frames after the last event, depending on the available data. Finally, they were stored in a new c3d file using BTK. These final c3d files are the ones reported in the present dataset.

## Data Records

All data records are available from figshare^24^. They are all stored in c3d file format (https://www.c3d.org). This file format is a public binary file format supported by all motion capture system manufacturers and biomechanics software programs. It is commonly used to store, for a single trial, synchronized 3D markers coordinates and analog data as well as a set of metadata (*e*.*g*. measurement units, custom parameters specific to the manufacturer software application).

Trial files are referenced in our dataset as YYYYNNN_CV_TT.c3d and static files as YYYYNNN_ST.c3d, organised by folder YYYYNNN, with:

- YYYY: year of the acquisition, *e*.*g*. 2014
- NNN: identification of the subject (passage number by year), *e*.*g*. 001
- CV: walking speed condition, *i*.*e*. C1, C2, C3, C4 or C5
- TT: trial number, *i*.*e*. 01 to 05

For each of the 50 participants, at least 3 trials (one right and one left gait cycle per trial) for each of the 5 conditions plus one static have been made available in the dataset, for a total of 1143 trials. Structure, labels, format, dimension, unit and description of each variable stored in the c3d files are given in Tables 1–4. Trial by trial information about the availability of forceplate data is given in Supplementary Table 2.

**Table 2:** Analog data stored in c3d files.

Labels	Format	Dim.	Unit	Description
R_tibialis_anterior	Real	m^*^ × 1	V	EMG^+^ signal of the right Tibialis Anterior
R_soleus	Real	m × 1	V	EMG signal of the right Soleus
R_gastrocnemius_medialis	Real	m × 1	V	EMG signal of the right Gastrocnemius Med.
R_vastus_medialis	Real	m × 1	V	EMG signal of the right Vastus Medialis
R_rectus_femoris	Real	m × 1	V	EMG signal of the right Rectus Femoris
R_semitendinosus	Real	m × 1	V	EMG signal of the right Semitendinosus
R_gluteus_maximus	Real	m × 1	V	EMG signal of the right Gluteus Maximus
R_gluteus_medius	Real	m × 1	V	EMG signal of the right Gluteus Medius
Fx1	Real	m × 1	N	Force applied by the foot on forceplate 1/X^¤^
Fy1	Real	m × 1	N	Force applied by the foot on forceplate 1/Y
Fz1	Real	m × 1	N	Force applied by the foot on forceplate 1/Z
Mx1	Real	m × 1	N.mm	Moment applied by the foot on forceplate 1/X
My1	Real	m × 1	N.mm	Moment applied by the foot on forceplate 1/Y
Mz1	Real	m × 1	N.mm	Moment applied by the foot on forceplate 1/Z
Fx2	Real	m × 1	N	Force applied by the foot on forceplate 2/X
Fy2	Real	m × 1	N	Force applied by the foot on forceplate 2/Y
Fz2	Real	m × 1	N	Force applied by the foot on forceplate 2/Z
Mx2	Real	m × 1	N.mm	Moment applied by the foot on forceplate 2/X
My2	Real	m × 1	N.mm	Moment applied by the foot on forceplate 2/Y
Mz2	Real	m × 1	N.mm	Moment applied by the foot on forceplate 2/Z

*Number of frames recorded at 1500 Hz.

^+^EMG: Electromyographic.

^¤^All forces and moments are expressed here in the coordinate system of the related forceplate (see Supplementary Fig. 1 for the coordinate system of each forceplate).

**Table 3:** Forceplate data stored in c3d files.

Structure	Labels	Format	Dim.	Unit	Description
ForcePlate(1)	P	Real	m* × 3	mm	Centre of pressure coordinates (forceplate 1)^¤^
	F	Real	m × 3	N	3D ground reaction force (forceplate 1)
	M	Real	m × 3	N.mm	3D ground reaction moment (forceplate 1)
ForcePlate(2)	P	Real	m* × 3	mm	Centre of pressure coordinates (forceplate 2)
	F	Real	m × 3	N	3D ground reaction force (forceplate 2)
	M	Real	m × 3	N.mm	3D ground reaction moment (forceplate 2)

*Number of frames recorded at 1500 Hz.

^¤^All centres of pressure, forces and moments are expressed here in the inertial coordinate system.

**Table 4:** Metadata* stored in c3d files.

Structure	Labels	Format	Dim.	Unit	Description
Subject	age	Integer	1 × 1	years	Age
	gender	Integer	1 × 1	none	0: woman, 1: man
	weight	Real	1 × 1	kg	Body weight
	height	Real	1 × 1	mm	Participant size
	R_legLength	Real	1 × 1	mm	Right leg length^+^
	L_legLength	Real	1 × 1	mm	Left leg length
Event	Right_Foot_Strike1	Real	1 × 1	s	First right foot strike timing
	Right_Foot_Strike2	Real	1 × 1	s	Second right foot strike timing
	Right_Foot_Off	Real	1 × 1 or 1 × 2	s	Right foot off timings
	Left_Foot_Strike1	Real	1 × 1	s	First left foot strike timing
	Left_Foot_Strike2	Real	1 × 1	s	Second left foot strike timing
	Left_Foot_Off	Real	1 × 1 or 1 × 2	s	Left foot off timings

*Additional metadata are stored by default (i.e. Copyright, Force_Platform, Point, Analog, Trial, Event_Context).

^+^Leg length is measured between the anterior-superior iliac spine and the medial tibial malleolus.

## Technical Validation

### Calibration of the optoelectronic system

As detailed in the procedure (see *Methods*), the optoelectronic system was calibrated before each session following the instructions available in the manufacturer’s documentation. In all calibration files, residuals (*i*.*e*. average of the different residuals of the 2D marker rays that belongs to the same 3D point) were below 2 mm, and the standard deviation of the reconstructed wand (*i*.*e*. calibration tool) length remained below 1.5 mm.

### 3D trajectories of cutaneous reflective markers

In all static and trial files, the 3D trajectories of cutaneous reflective markers were fully reconstructed (*i*.*e*. 0% of gap in the trajectories), and residuals remained below 4 mm.

### Centre of pressure location

The accuracy of the centre of pressure location was not specifically assessed during these data records. However, the accuracy of the centre of pressure location has previously been estimated using the Caltester procedure (Visual 3D v6, C-Motion, USA) to 3.11 ± 0.69 mm along X axis, 0.98 ± 0.54 mm along the Y axis and 1.55 ± 0.11 along the Z axis for forceplate 1, 3.56 ± 0.89 mm along X axis, 3.10 ± 0.79 mm along the Y axis and 1.70 ± 0.12 along the Z axis for forceplate 2.

## Usage Notes

The data records stored in c3d file format (https://www.c3d.org) can easily be read using c3d parsers such as the Biomechanics ToolKit (BTK) (http://biomechanical-toolkit.github.io/)^22^ and the ezc3d package (https://github.com/pyomeca/ezc3d). The Motion kinematic and kinetic analyzer (Mokka) can also be a convenient tool for 3D visualisation (http://biomechanical-toolkit.github.io/mokka/index.html). Anthropometric and demographic parameters of each participant are stored in the metadata of the related c3d files. Based on the markerset used in this study, joint kinematics and dynamics can be computed using the 3D Kinematics and Inverse Dynamics toolbox proposed by Dumas and freely available on the MathWorks File Exchange (https://nl.mathworks.com/matlabcentral/fileexchange/58021-3d-kinematics-and-inverse-dynamics).

## Supplementary Information

### ISA-Tab metadata file

Download metadata file.

### Supplementary information

Supplementary materials.

## Data Availability

The custom Matlab code used to process data (see previous section) is freely available on the following repository: https://github.com/fmoissenet/CGA_Rehazenter_Toolbox/tree/article_ScientificData2019. The Biomechanics ToolKit (BTK) is also freely available on the following repository: http://biomechanical-toolkit.github.io/.

## Online-only Table

**Online-only Table 1:** Anthropometric and demographic information of the participants.

Subject ID	Gender	Age (year)	Height (m)	Mass (Kg)	BMI (kg.m^−2^)	Right leg length (m)	Left leg length (m)
2014001	M	31	1.66	67.0	24.5	0.731	0.735
2014002	W	48	1.64	65.4	24.3	0.774	0.770
2014003	W	28	1.56	50.0	20.5	0.721	0.720
2014004	M	23	1.77	72.5	23.4	0.829	0.848
2014005	M	25	1.83	73.5	21.9	0.864	0.892
2014006	M	23	1.76	73.0	23.6	0.849	0.856
2014007	W	44	1.69	65.0	22.8	0.837	0.838
2014008	W	30	1.66	57.1	20.7	0.802	0.793
2014009	M	57	1.88	86.0	24.3	0.897	0.890
2014011	M	59	1.80	63.4	19.6	0.849	0.854
2014013	W	26	1.70	61.3	21.2	0.789	0.784
2014014	M	29	1.80	92.0	28.4	0.842	0.847
2014015	W	22	1.58	67.0	26.8	0.716	0.708
2014019	W	26	1.76	73.8	24.0	0.819	0.828
2014022	W	48	1.71	59.8	20.5	0.819	0.826
2014024	M	33	1.92	87.5	23.7	0.906	0.906
2014025	W	31	1.66	80.5	29.2	0.781	0.788
2014029	M	38	1.89	89.9	25.3	0.877	0.885
2014030	W	62	1.70	60.7	21.0	0.802	0.806
2014031	M	21	1.77	67.2	21.4	0.802	0.822
2014033	W	24	1.60	63.5	24.8	0.706	0.714
2014034	M	21	1.84	89.6	26.5	0.902	0.899
2014040	W	19	1.55	56.5	23.5	0.734	0.715
2014046	W	40	1.65	61.8	22.7	0.850	0.853
2014048	W	40	1.64	61.5	22.9	0.810	0.816
2014049	M	32	1.74	72.2	23.8	0.841	0.835
2014050	W	28	1.64	61.9	23.0	0.750	0.758
2014051	M	25	1.91	88.0	24.1	0.920	0.915
2014052	M	25	1.82	79.5	24.0	0.871	0.858
2014053	W	21	1.72	62.8	21.2	0.835	0.822
2015002	M	39	1.74	74.0	24.4	0.828	0.838
2015003	M	52	1.77	87.2	27.8	0.843	0.847
2015004	W	35	1.70	62.0	21.5	0.802	0.809
2015005	M	48	1.90	89.4	24.8	0.874	0.877
2015007	W	63	1.66	60.2	21.8	0.755	0.752
2015013	W	58	1.69	73.0	25.6	0.808	0.808
2015015	W	50	1.73	68.0	22.7	0.816	0.829
2015016	W	46	1.69	76.0	26.6	0.855	0.829
2015017	W	41	1.67	60.5	21.7	0.805	0.806
2015020	M	43	1.79	95.0	29.6	0.845	0.846
2015021	W	30	1.69	58.0	20.3	0.775	0.788
2015026	W	64	1.71	51.5	17.7	0.800	0.814
2015027	M	51	1.72	65.5	22.1	0.791	0.788
2015030	M	24	1.87	86.0	24.6	0.917	0.917
2015032	M	26	1.72	50.8	17.2	0.803	0.817
2015035	M	38	1.77	81.5	26.0	0.819	0.839
2015037	M	42	1.76	66.1	21.3	0.837	0.850
2015041	M	31	1.88	74.8	21.2	0.876	0.857
2015042	M	67	1.83	98.0	29.3	0.855	0.874
2015043	M	21	1.78	74.0	23.4	0.840	0.833
