# Supplementary material for: A multimodal dataset of human gait at different walking speeds established on injury-free adult participants
Source: Sci Data. 2019 Jul 3;6:111. doi: 10.1038/s41597-019-0124-4 (PMC6610108; doi:10.1038/s41597-019-0124-4)

# Supplementary data

**Supplementary table 1** – Participant’s health condition and sports habits

| **Subject Id** | **Q1** | **Q2** | **Q3** | **Q4** | **Q5** | **Q6** | **Q7** | **Q8** | **Q9** |
| --- | --- | --- | --- | --- | --- | --- | --- | --- | --- |
| 2014001 | S | 3h<…<5h | running, crossfit | NA | NA | NA | NA | NA | NA |
| 2014002 | S | <1h | NA | NA | NA | NA | NA | NA | NA |
| 2014003 | P | 1h<…<3h | running, capoeira, | NA | NA | NA | NA | NA | NA |
| 2014004 | P | >5h | running, soccer, badminton, | NA | RA | RF | T | NA | NA |
| 2014005 | S | 3h<…<5h | running, biking | NA | RK | NA | NA | NA | NA |
| 2014006 | P | 1h<…<3h | fitness | NA | RA+LA | NA | NA | P | NA |
| 2014007 | S | <1h | NA | NA | NA | NA | L | C | NA |
| 2014008 | P | <1h | NA | NA | NA | NA | L | C | NA |
| 2014009 | P | 3h<…<5h | biking | NA | A | NA | NA | NA | Right LCA |
| 2014011 | S | >5h | running, walking, biking | NA | NA | NA | NA | NA | NA |
| 2014013 | P | >5h | basket | NA | RA | NA | NA | NA | NA |
| 2014014 | P | >5h | Running, bodybuilding, rugby, crossfit, squash | NA | RA+LA | NA | NA | NA | NA |
| 2014015 | S | 1h<…<3h | cardio-training | NA | NA | NA | NA | NA | NA |
| 2014019 | P | 1h<…<3h | running, swimming | NA | NA | NA | NA | NA | NA |
| 2014022 | P | 1h<…<3h | cardio-training | NA | LH | NA | L | NA | NA |
| 2014024 | P | >5h | running, biking, volleyball | NA | RA+LA+RK | RA | T | P | NA |
| 2014025 | S | 1h<…<3h | swimming, cardio-training | NA | RK | NA | NA | NA | Left leg denervation (2011) |
| 2014029 | P | >5h | swimming, bodybuilding | NA | RK | NA | NA | NA | Right LCA (2001 and 2012) |
| 2014030 | P | >5h | walking, soft gymnastics | RH+LH | LH | NA | TL | C+R | Hernia L4-L5 (2007) |
| 2014031 | P | 1h<…<3h | running | NA | NA | NA | NA | NA | NA |
| 2014033 | S | 1h<…<3h | cardio-training | NA | NA | NA | TL | NA | NA |
| 2014034 | P | <1h | running, swimming | NA | NA | NA | NA | P | NA |
| 2014040 |  | 1h<…<3h | various | NA | NA | NA | NA | P | NA |
| 2014046 | P | 3h<…<5h | horse riding, aquagym | NA | NA | NA | NA | NA | NA |
| 2014048 |  | 1h<…<3h | horse riding | NA | NA | NA | NA | NA | NA |
| 2014049 | P | >5h | running | NA | NA | NA | NA | NA | NA |
| 2014050 | P | 3h<…<5h | biking, walking, swimming | NA | RA | RF | NA | NA | NA |
| 2014051 | S | 1h<…<3h | biking, badminton | NA | RK | NA | NA | NA | NA |
| 2014052 | P | 1h<…<3h | foot, running | NA | RA+LA+ RK | NA | NA | P | NA |
| 2014053 | P | >5h | fitness, running | NA | NA | NA | NA | P | NA |
| 2015002 | S | 3h<…<5h | biking, running | NA | RK+LK | NA | NA | NA | LCA (2004 and 2013) |
| 2015003 | P | <1h | NA | NA | NA | NA | NA | NA | NA |
| 2015004 | P | 1h<…<3h | crossfit | NA | NA | NA | NA | P | NA |
| 2015005 | P | >5h | biking, swimming, running | NA | NA | NA | NA | NA | NA |
| 2015007 | S | 1h<…<3h | tennis, golf, soft gymnastics | NA | LK | RF | T | P | NA |
| 2015013 | P | 3h<…<5h | walking, soft gymnastics | NA | LA+RA | NA | NA | NA | NA |
| 2015015 | P | 3h<…<5h | running, soft gymnastics | NA | NA | NA | NA | NA | NA |
| 2015016 | P | 1h<…<3h | running, horse riding | NA | RA+LA | NA | NA | NA | NA |
| 2015017 | P | <1h | running | NA | NA | NA | NA | NA | NA |
| 2015020 | S | >5h | biking, running, swimming, volleyball | NA | NA | NA | NA | NA | NA |
| 2015021 | P | 3h<…<5h | running, cardio, fitness | NA | NA | NA | NA | P | NA |
| 2015026 | S | 1h<…<3h | swimming, walking | NA | NA | RF | NA | NA | NA |
| 2015027 | P | 1h<…<3h | walking, biking | NA | LK | NA | NA | NA | Left LCA (2003) |
| 2015030 | S | >5h | fottball, running | NA | LA+LK | NA | NA | NA | NA |
| 2015032 | S | 3h<…<5h | climbing, fitness |  |  |  |  |  |  |
| 2015035 | S | <1h | NA | NA | RA | RT | NA | NA | NA |
| 2015037 | S | <1h | NA | NA | NA | RF | NA | NA | NA |
| 2015041 | P | <1h | NA | NA | NA | RF | NA | NA | NA |
| 2015042 | NA | 1h<…<3h | bodybuilding, cardio | NA | NA | RF | NA | P | NA |
| 2015043 | P | >5h | biking | NA | RA | RF | NA | NA | NA |

***Q1****: Occupation (S: sedentary, P: physical) -* ***Q2****: Sports (hours per week) -* ***Q3****: Sport -* ***Q4****: Prosthesis (R: right, L: left, H: hip, K: knee, A: ankle, S: shoulder) -* ***Q5****: Musculo-tendino-ligamentous lesion -* ***Q6****: Fractures (P: pelvis, F: femur, T: tibia/fibula, F: foot) -* ***Q7****: Scoliosis (T: thoracic, L: lumbar) -* ***Q8****: Low back pain (P: punctate, C: chronic, R: radiating) -* ***Q9****: Orthopedic operations - NA: not applicable*

**Supplementary table 2** – Available ground reaction forces and moments across participants and trials (NA: trial not available). The first number corresponds to the right foot, the second to the left foot. The forceplate number is given to indicate which ground reaction forces and moments are applied on the related foot (1: forceplate(1), 2: forceplate(2), 0: ground reaction information not available or not reliable).

|  |  |  |  |  | **Trial files for each walking speed condition** | | | | | | | | | | | | | | | | | | | | | | | | | |  |
| --- | --- | --- | --- | --- | --- | --- | --- | --- | --- | --- | --- | --- | --- | --- | --- | --- | --- | --- | --- | --- | --- | --- | --- | --- | --- | --- | --- | --- | --- | --- | --- |
|  | **C1** | | | | | | **C2** | | | | | | **C3** | | | | | | **C4** | | | | | | **C5** | | | | | | |
| **Subject Id** | 01 | 02 | 03 | 04 | 05 | 06 | 01 | 02 | 03 | 04 | 05 | 06 | 01 | 02 | 03 | 04 | 05 | 06 | 01 | 02 | 03 | 04 | 05 | 06 | 01 | 02 | 03 | 04 | 05 | 06 | |
| 2014001 | 12 | 21 | 21 | 21 | 21 | NA | 12 | 01 | 12 | 21 | 21 | NA | 21 | 21 | 12 | 01 | 12 | NA | 01 | 21 | 01 | 12 | 21 | NA | 21 | 20 | 01 | 10 | 20 | NA | |
| 2014002 | 10 | 20 | 10 | 20 | 10 | NA | 12 | 01 | 12 | NA | NA | NA | 12 | 12 | 21 | NA | NA | NA | 10 | 21 | 10 | 21 | NA | NA | 02 | 10 | 02 | 02 | 10 | NA | |
| 2014003 | 01 | 02 | 01 | 01 | 02 | NA | 02 | 01 | 02 | 01 | 02 | NA | 12 | 12 | 12 | NA | NA | NA | 21 | 10 | 21 | NA | NA | NA | 10 | 02 | 10 | 10 | 10 | 01 | |
| 2014004 | 02 | 02 | 10 | 01 | 10 | NA | 21 | 21 | 12 | 12 | 00 | NA | 10 | 21 | 12 | 21 | NA | NA | 01 | 12 | 01 | 02 | NA | NA | 12 | 20 | 01 | 02 | 01 | NA | |
| 2014005 | 01 | 02 | 10 | 20 | 02 | NA | 12 | 21 | 21 | 21 | 12 | NA | 12 | 12 | 12 | 21 | 12 | NA | 12 | 12 | 12 | 21 | 20 | NA | 10 | 20 | 10 | 20 | NA | NA | |
| 2014006 | 02 | 10 | 10 | 21 | NA | NA | 12 | 12 | 21 | 12 | NA | NA | 10 | 10 | 10 | 10 | 21 | NA | 01 | 10 | 01 | 12 | NA | NA | 01 | 01 | 01 | 20 | 01 | NA | |
| 2014007 | 02 | 01 | 01 | 01 | 02 | NA | 12 | 12 | 12 | 02 | 02 | NA | 12 | 12 | 21 | 12 | NA | NA | 12 | 21 | 12 | 21 | 21 | NA | 21 | 12 | 21 | 12 | NA | NA | |
| 2014008 | 02 | 01 | 02 | 20 | 02 | NA | 12 | 00 | 12 | 12 | 21 | NA | 12 | 20 | 12 | 21 | 12 | NA | 01 | 12 | 12 | 01 | 21 | NA | 20 | 10 | 10 | 02 | NA | NA | |
| 2014009 | 01 | 01 | 01 | 20 | 01 | NA | 12 | 12 | 21 | 12 | NA | NA | 10 | 01 | 20 | 10 | 12 | NA | 10 | 10 | 10 | 20 | 12 | NA | 01 | 02 | 20 | 01 | 01 | NA | |
| 2014011 | 10 | 02 | 20 | 02 | 10 | NA | 21 | 12 | 21 | 12 | NA | NA | 21 | 12 | 21 | 12 | NA | NA | 21 | 12 | 20 | 20 | NA | NA | 01 | 01 | 01 | 01 | 20 | NA | |
| 2014013 | 10 | 01 | 10 | 10 | NA | NA | 21 | 21 | 10 | 21 | 12 | NA | 12 | 01 | 12 | 21 | NA | NA | 12 | 21 | 12 | 21 | NA | NA | 20 | 02 | 10 | 02 | 02 | NA | |
| 2014014 | 02 | 10 | 01 | 10 | 20 | NA | 20 | 21 | 12 | 21 | NA | NA | 21 | 10 | 21 | 20 | 12 | NA | 21 | 21 | 12 | 21 | 12 | NA | 12 | 12 | 20 | 12 | NA | NA | |
| 2014015 | 01 | 20 | 02 | 20 | 01 | NA | 12 | 21 | 12 | 21 | 12 | NA | 21 | 21 | 21 | 21 | NA | NA | 10 | 21 | 10 | 21 | 12 | NA | 01 | 01 | 21 | 10 | 01 | NA | |
| 2014019 | 01 | 10 | 20 | 02 | 02 | NA | 21 | 02 | 00 | 00 | 21 | NA | 12 | 21 | 12 | 12 | 10 | NA | 12 | 21 | 12 | 21 | 12 | NA | 20 | 10 | 12 | 02 | 21 | NA | |
| 2014022 | 01 | 01 | 02 | 01 | 02 | NA | 12 | 21 | 21 | 12 | 21 | NA | 21 | 12 | 21 | 12 | NA | NA | 12 | 02 | 21 | 12 | 01 | NA | 01 | 21 | 20 | NA | NA | NA | |
| 2014024 | 10 | 10 | 02 | 20 | 20 | NA | 21 | 12 | 21 | 12 | 21 | NA | 21 | 12 | 12 | 21 | 12 | NA | 20 | 10 | 01 | 10 | 01 | NA | 20 | 02 | 10 | 20 | 02 | NA | |
| 2014025 | 02 | 01 | 01 | 01 | 02 | NA | 21 | 12 | 01 | 12 | 21 | NA | 12 | 21 | 12 | 20 | 20 | NA | 02 | 12 | 12 | 12 | NA | NA | 10 | 21 | 12 | 02 | NA | NA | |
| 2014029 | 01 | 10 | 01 | 01 | 10 | NA | 10 | 21 | 21 | 12 | 21 | NA | 20 | 10 | 21 | 20 | 12 | NA | 10 | 21 | 12 | 21 | 21 | NA | 12 | 21 | 01 | 12 | 21 | NA | |
| 2014030 | 01 | 02 | 02 | 01 | 01 | NA | 12 | 12 | 12 | NA | NA | NA | 21 | 21 | 12 | 12 | 12 | NA | 12 | 21 | 12 | 12 | 21 | NA | 12 | 12 | 02 | 12 | 02 | NA | |
| 2014031 | 02 | 01 | 02 | 01 | 02 | NA | 10 | 12 | 12 | 21 | NA | NA | 21 | 10 | 21 | 21 | NA | NA | 10 | 21 | 12 | 21 | 12 | NA | 02 | 20 | 10 | 10 | 21 | NA | |
| 2014033 | NA | 21 | 01 | 20 | NA | NA | 12 | 21 | 12 | 10 | NA | NA | 21 | 01 | 12 | NA | NA | NA | 02 | 21 | 12 | 20 | 21 | NA | 10 | 10 | 02 | 02 | NA | NA | |
| 2014034 | 02 | 20 | 10 | 20 | 01 | NA | 12 | 20 | 10 | 21 | 21 | NA | 12 | 01 | 01 | NA | NA | NA | 20 | 01 | 02 | 01 | 12 | NA | 02 | 10 | 10 | NA | NA | NA | |
| 2014040 | 10 | 10 | 10 | 20 | 10 | NA | 01 | 20 | 10 | 01 | 01 | NA | 21 | 12 | 21 | 12 | 21 | NA | 21 | 12 | 21 | 21 | NA | NA | 20 | 10 | 20 | 10 | 01 | NA | |
| 2014046 | 02 | 20 | 02 | 10 | 10 | NA | 12 | 12 | 20 | 10 | 21 | NA | 10 | 10 | 12 | 02 | NA | NA | 01 | 12 | 01 | 02 | 01 | NA | 02 | 10 | 02 | 01 | 10 | NA | |
| 2014048 | 20 | 01 | 10 | 02 | 10 | NA | 02 | 20 | 01 | 01 | 10 | NA | 01 | 12 | 21 | 20 | NA | NA | 12 | 21 | 12 | 21 | 12 | NA | 02 | 21 | 20 | 02 | 12 | NA | |
| 2014049 | 01 | 02 | 01 | 01 | 02 | NA | 12 | 12 | 12 | 21 | 12 | NA | 12 | 21 | 21 | 12 | NA | NA | 20 | 12 | 01 | 12 | 21 | NA | 20 | 02 | 10 | 02 | 20 | NA | |
| 2014050 | 02 | 02 | 01 | 01 | 01 | NA | 01 | 01 | 01 | 01 | 02 | NA | 12 | 21 | 12 | 21 | 12 | NA | 01 | 02 | 10 | 12 | 01 | NA | 02 | 02 | 20 | 02 | 21 | NA | |
| 2014051 | 12 | 21 | 12 | 21 | NA | NA | 12 | 21 | 12 | 12 | NA | NA | 21 | 12 | 21 | 12 | 21 | NA | 21 | 12 | 12 | 21 | 21 | NA | 01 | 20 | 10 | 10 | NA | NA | |
| 2014052 | 10 | 01 | 02 | 20 | 01 | NA | 21 | 21 | 21 | NA | NA | NA | 02 | 12 | 12 | NA | NA | NA | 02 | 01 | 21 | 02 | NA | NA | 02 | 10 | 10 | NA | NA | NA | |
| 2014053 | 10 | 10 | 20 | 01 | 01 | NA | 21 | 21 | 12 | 21 | 12 | NA | 02 | 20 | 12 | 01 | 01 | NA | 21 | 12 | 12 | 21 | 12 | NA | 10 | 01 | 10 | 10 | 10 | NA | |
| 2015002 | 12 | 12 | 02 | 21 | 12 | NA | 01 | 21 | 12 | 21 | 12 | NA | 12 | 12 | 21 | 21 | 21 | NA | 12 | 21 | 10 | 21 | 01 | NA | 01 | 10 | 10 | 10 | NA | NA | |
| 2015003 | 02 | 10 | 02 | 20 | 10 | NA | 01 | 01 | 21 | 02 | 12 | NA | 01 | 10 | 21 | 21 | 10 | NA | 12 | 02 | 21 | 21 | NA | NA | 02 | 10 | 10 | 01 | 20 | NA | |
| 2015004 | 20 | 12 | 21 | 12 | 21 | NA | 12 | 21 | 10 | 21 | 21 | NA | 20 | 21 | 12 | 21 | 10 | NA | 12 | 01 | 02 | 12 | 21 | NA | 20 | 20 | 01 | 20 | 10 | NA | |
| 2015005 | 10 | 10 | 02 | 01 | 02 | NA | 02 | 01 | 02 | 02 | 20 | NA | 02 | 20 | 21 | 21 | 01 | NA | 01 | 12 | 10 | 20 | 10 | NA | 10 | 01 | 02 | 02 | 02 | NA | |
| 2015007 | 10 | 10 | 02 | 10 | 02 | NA | 12 | 10 | 10 | 10 | NA | NA | 21 | 12 | 02 | 12 | 12 | NA | 02 | 12 | 21 | 12 | 21 | NA | 20 | 01 | 12 | NA | NA | NA | |
| 2015013 | 02 | 01 | 02 | 01 | 01 | NA | 21 | 12 | 12 | 21 | 12 | NA | 10 | 20 | 10 | 21 | 21 | NA | 21 | 02 | 01 | 02 | 02 | NA | 01 | 02 | 20 | 01 | 02 | NA | |
| 2015015 | 21 | 21 | 21 | NA | NA | NA | 21 | 21 | 12 | 20 | NA | NA | 20 | 10 | 10 | 20 | 21 | NA | 20 | 21 | 10 | 21 | 12 | NA | 02 | 01 | 01 | 10 | 01 | NA | |
| 2015016 | 01 | 02 | 02 | 02 | 02 | NA | 12 | 12 | 00 | NA | NA | NA | 21 | 21 | 21 | 12 | NA | NA | 10 | 21 | 12 | 01 | NA | NA | 02 | 02 | 10 | 10 | NA | NA | |
| 2015017 | 02 | 01 | 02 | 01 | 01 | NA | 10 | 02 | 01 | 02 | 01 | NA | 21 | 12 | 21 | 12 | 21 | NA | 20 | 20 | 10 | 20 | 10 | NA | 21 | 01 | 20 | 20 | 10 | NA | |
| 2015020 | 01 | 20 | 20 | 20 | 02 | NA | 12 | 20 | 12 | 21 | 21 | NA | 10 | 12 | 20 | 01 | 02 | NA | 12 | 21 | 21 | 02 | 12 | NA | 20 | 20 | 01 | 10 | 10 | NA | |
| 2015021 | 01 | 01 | 20 | 20 | 02 | NA | 10 | 02 | 20 | 01 | 10 | NA | 12 | 01 | 12 | 12 | NA | NA | 10 | 01 | 10 | 21 | 12 | NA | 20 | 10 | 10 | 10 | NA | NA | |
| 2015026 | 20 | 20 | 01 | 01 | NA | NA | 02 | 10 | 02 | 20 | 02 | NA | 12 | 12 | 12 | 20 | NA | NA | 21 | 20 | 20 | 21 | NA | NA | 01 | 02 | 21 | NA | NA | NA | |
| 2015027 | 10 | 01 | 01 | 20 | NA | NA | 02 | 20 | 10 | 10 | 01 | NA | 21 | 12 | 21 | 20 | 12 | NA | 10 | 12 | 21 | 21 | NA | NA | 20 | 02 | 20 | 10 | NA | NA | |
| 2015030 | 01 | 01 | 01 | 01 | 10 | NA | 20 | 01 | 01 | 20 | 12 | NA | 10 | 21 | 01 | NA | NA | NA | 20 | 21 | 20 | 21 | 12 | NA | 02 | 20 | 01 | NA | NA | NA | |
| 2015032 | 20 | 01 | 01 | 10 | 01 | NA | 12 | 12 | 12 | 21 | NA | NA | 21 | 12 | 12 | 21 | NA | NA | 12 | 21 | 12 | NA | NA | NA | 21 | 02 | 01 | 02 | 01 | NA | |
| 2015035 | 10 | 10 | 10 | 10 | 01 | NA | 02 | 12 | 01 | 12 | NA | NA | 10 | 02 | 01 | 20 | 10 | NA | 21 | 10 | 21 | 10 | 20 | NA | 01 | 02 | 21 | NA | NA | NA | |
| 2015037 | 20 | 01 | 10 | 02 | 10 | NA | 12 | 21 | 20 | NA | NA | NA | 01 | 02 | 21 | 10 | 12 | NA | 12 | 12 | 01 | 12 | NA | NA | 10 | 10 | 10 | NA | NA | NA | |
| 2015041 | 02 | 12 | 12 | 12 | NA | NA | 12 | 12 | 21 | 21 | 12 | NA | 20 | 12 | 10 | 12 | NA | NA | 02 | 10 | 20 | 20 | NA | NA | 10 | 10 | 10 | 10 | NA | NA | |
| 2015042 | 20 | 01 | 01 | 01 | 01 | NA | 21 | 21 | 12 | 21 | NA | NA | 21 | 12 | 20 | 12 | 10 | NA | 20 | 10 | 10 | 20 | NA | NA | 01 | 02 | 20 | 01 | 10 | NA | |
| 2015043 | 02 | 20 | 20 | 20 | 02 | NA | 21 | 01 | 12 | 21 | 12 | NA | 12 | 21 | 12 | 21 | NA | NA | 21 | 10 | 10 | 12 | 21 | NA | 21 | 21 | 01 | 21 | 21 | NA | |

**Supplementary figure 1** – Inertial coordinate system and forceplates coordinate systems used in this dataset


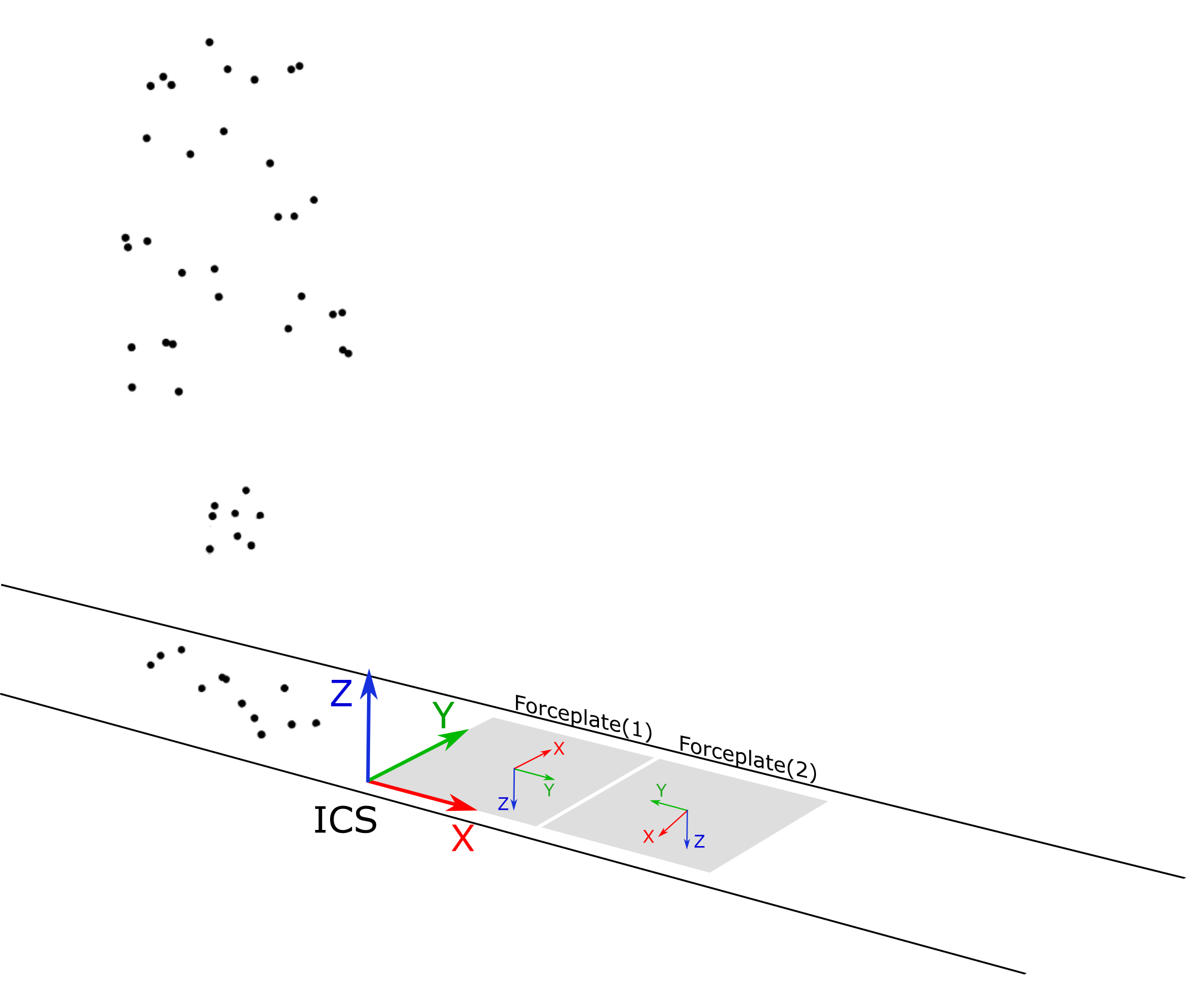

Supplement: Supplementary file 2 — Supplementary materials. [file 41597_2019_124_MOESM2_ESM.docx]
